# Population pharmacokinetic and pharmacokinetic-pharmacodynamic modeling of bempedoic acid and low-density lipoprotein cholesterol in healthy subjects and patients with dyslipidemia

**DOI:** 10.1007/s10928-023-09864-w

**Published:** 2023-05-27

**Authors:** Satyawan B. Jadhav, Benny M. Amore, Howard Bockbrader, Ryan L. Crass, Sunny Chapel, William J. Sasiela, Maurice G. Emery

**Affiliations:** 1 Ann Arbor Pharmacometrics Group, 900 Victors Way #328, Ann Arbor, MI 48108 USA; 2 Esperion Therapeutics, Inc., 3891 Ranchero Drive, Suite 150, Ann Arbor, MI 48108 USA

**Keywords:** Bempedoic acid, Hypercholesterolemia, Low-density lipoprotein cholesterol, Pharmacodynamics, Pharmacokinetics, Exposure–response

## Abstract

Population pharmacokinetics (popPK) of bempedoic acid and the popPK/pharmacodynamic (popPK/PD) relationship between bempedoic acid concentrations and serum low-density lipoprotein cholesterol (LDL-C) from baseline were characterized. A two-compartment disposition model with a transit absorption compartment and linear elimination best described bempedoic acid oral pharmacokinetics (PK). Multiple covariates, including renal function, sex, and weight, had statistically significant effects on the predicted steady-state area under the curve. Mild (estimated glomerular filtration rate (eGFR) 60 to < 90 mL/min vs. ≥ 90 mL/min) and moderate (eGFR 30 to < 60 mL/min vs. ≥ 90 mL/min) renal impairment, female sex, low (< 70 kg vs. 70–100 kg) and high (> 100 kg vs. 70–100 kg) body weight were predicted to have a 1.36-fold (90% confidence interval (CI) 1.32, 1.41), 1.85-fold (90% CI 1.74, 2.00), 1.39-fold (90% CI 1.34, 1.47), 1.35-fold (90% CI 1.30, 1.41), and 0.75-fold (90% CI 0.72, 0.79) exposure difference relative to their reference populations, respectively. An indirect response model described changes in serum LDL-C with a model-predicted 35% maximal reduction and bempedoic acid IC_50_ of 3.17 µg/mL. A 28% reduction from LDL-C baseline was predicted for a steady-state average concentration of 12.5 µg/mL after bempedoic acid (180 mg/day) dosing, accounting for approximately 80% of the predicted maximal LDL-C reduction. Concurrent statin therapy, regardless of intensity, reduced the maximal effect of bempedoic acid but resulted in similar steady-state LDL-C levels. While multiple covariates had statistically significant effects on PK and LDL-C lowering, none were predicted to warrant bempedoic acid dose adjustment.

**Supplementary Information:**

The online version contains supplementary material available at 10.1007/s10928-023-09864-w.

## Introduction

Alterations in lipid and lipoprotein metabolism play an important role in the pathogenesis of atherosclerotic cardiovascular disease (ASCVD), and reductions in low-density lipoprotein cholesterol (LDL-C) represent a primary therapeutic target for primary and secondary prevention of ASCVD events [1, 2]. Statins are an established first-line option, with the potential addition of ezetimibe or proprotein convertase subtilisin/kexin type 9 (PCSK9) inhibitors for lowering LDL-C and reducing cardiovascular risk. The 2018 joint American College of Cardiology/American Heart Association guidelines advocate the use of high-intensity or maximally tolerated statin therapy, with or without the addition of non-statin agents [1]. However, treatment options are limited for patients who cannot take statins or those who do not achieve LDL-C goals despite maximally tolerated statin therapy; these patients remain at elevated cardiovascular risk due to persistently elevated LDL-C [3, 4].

Bempedoic acid is an oral, once-daily medication that lowers LDL-C in patients with hypercholesterolemia, ASCVD, and/or heterozygous familial hypercholesterolemia (HeFH) [5–9]. Bempedoic acid is a prodrug converted to the pharmacologically active bempedoyl-coenzyme A (CoA) ester by very long-chain acyl-CoA synthetase 1 (ACSVL1, SLC27A2) activity localized in human hepatocytes. Bempedoyl-CoA is a potent and selective competitive inhibitor of adenosine triphosphate (ATP)-citrate lyase, a cytosolic enzyme proximal to 3-hydroxy-3-methylglutaryl-CoA (HMG-CoA) reductase in the lipid biosynthesis pathway, catalyzing the formation of oxaloacetate and acetyl-CoA from mitochondrial-derived citrate. Inhibition of ATP-citrate lyase by bempedoyl-CoA results in LDL-C lowering by decreasing cholesterol synthesis and upregulating LDL receptors [10, 11]. As ACSVL1 is mainly present in the liver and not in skeletal muscle [10], it is hypothesized that the risk of muscle-related adverse effects is lower with bempedoic acid compared with statins. Across phase 3 studies conducted during clinical development, rates of myalgia and muscle weakness among patients receiving bempedoic acid were comparable with placebo, consistent with the proposed hypothesis [12].

The pharmacokinetics (PK) of bempedoic acid is typically characterized by maximum plasma concentrations at approximately 3.5 h after dosing, a mean (standard deviation (SD)) elimination half-life of 21 (11) h, and steady-state concentrations achieved after 7 days following once-daily 180 mg dose administration. Following a single administration of radiolabeled bempedoic acid 240 mg orally, approximately 70% of the total dose was recovered in urine, primarily as bempedoic acid acyl glucuronide, and approximately 30% was recovered in feces; less than 5% of the administered dose was recovered as unchanged parent drug in urine and feces combined [11].

Patients with hypercholesterolemia, who frequently receive lipid-modifying therapies (LMTs), constitute a diverse population with differing comorbid and potential polypharmacy conditions. To clarify the complex relationship between bempedoic acid plasma exposures and serum LDL-C response in a diverse patient population, including in patients receiving other LMTs, population PK (popPK) and pharmacodynamic (PD) models were developed in a pooled analysis of phase 1, 2, and 3 studies conducted during the clinical development of bempedoic acid. The primary aims of this investigation were to: (1) characterize plasma PK of bempedoic acid and PK/PD relationships between bempedoic acid plasma concentrations and serum LDL-C lowering in healthy subjects and patients with hypercholesterolemia, ASCVD, and/or HeFH, (2) estimate the intra- and interindividual variability in plasma PK and serum LDL-C lowering and derive quantitative predictions of bempedoic acid exposure and the resulting LDL-C response, and (3) examine the effects of intrinsic and extrinsic factors to determine potential sources of PK and PD variability.

## Methods

### Clinical studies

The popPK analysis used pooled bempedoic acid dose–plasma concentration vs. time data from 22 clinical studies (nine phase 1, nine phase 2, and four phase 3 studies) that included 2232 healthy subjects and patients with dyslipidemia, renal or hepatic impairment, or type 2 diabetes mellitus [6–8, 13–23]. The population PK/PD (popPK/PD) analysis included LDL-C response data from 4459 patients in bempedoic acid–treated and placebo treatment groups from 15 clinical studies (three phase 1, eight phase 2, and four phase 3 studies), representing a subset of the 22 studies used in the popPK analysis. Placebo-treated study participants were included into the popPK/PD model as having a bempedoic acid concentration of 0 µg/mL. Pivotal phase 3 studies were double-blind, randomized, placebo-controlled studies of oral bempedoic acid 180 mg/day used either alone or in combination with a stable background of LMTs. Two phase 3 studies included patients with elevated LDL-C and prior ASCVD and/or HeFH who were receiving maximally tolerated statin therapy [6, 8]. The other phase 3 studies included patients with elevated LDL-C and varying degrees of cardiovascular risk who had a history of statin intolerance and received bempedoic acid either alone or concomitantly with a low-intensity statin or other LMTs, such as ezetimibe [7, 13]. Details of the individual studies are summarized in Online Resource 1.

### Software

Analyses were performed using the nonlinear mixed-effects modeling methodology as implemented in NONMEM® software (version 7.3). Data post-processing, including graphical analysis, was performed using SAS (version 9.3 or 9.4) or R (version 3.1.2 or higher).

### Bioanalytical methods

Bempedoic acid plasma concentrations were analyzed by liquid chromatography-tandem mass spectrometry [24]. The lower limit of quantification for bempedoic acid ranged from 0.01 to 0.02 μg/mL across studies.

### PopPK analysis

#### Base structural model development

A base structural model was initially developed using pooled data from 10 clinical studies (nine phase 1 and one phase 2 trials), with intensive PK sampling following oral bempedoic acid doses of 60–240 mg in healthy subjects, patients with hyperlipidemia, or those with impaired renal or hepatic function. PK data from study participants who received ≥ 80% of their planned dose regimen (per recorded pill counts) were included.

To characterize bempedoic acid PK following single- and multiple-dose oral administration, structural model development began with the evaluation of a one-compartment model with first-order absorption and elimination. Absolute bioavailability was not identifiable in the absence of an intravenous reference formulation, and PK parameters of systemic clearance (CL) and central distribution volume (Vc) were interpreted as their apparent values after oral dose administration. Additional structural models were assessed to evaluate further model complexities including multiple distribution compartments, alternate absorption models (time-lagged, zero-order, parallel zero-order and first-order, transit), and nonlinear elimination. The PK of ESP15228, a metabolite of bempedoic acid which is further metabolized by ACSVL1 to an approximately equipotent pharmacologically active CoA ester, was also assessed for inclusion. While a structural model that included ESP15228 was evaluated, the model was simplified to characterize bempedoic acid PK alone, as ESP15228 circulates in plasma at a constant proportion of parent drug concentrations (approximately 20%) under a variety of clinically relevant conditions and its formation represents a relatively minor pathway of bempedoic acid metabolic clearance. This suggested that the effects of bempedoic acid are well characterized by parent drug concentrations and that parent drug can be considered a surrogate measure of LDL-C lowering by bempedoic acid and its active metabolite. The final model structure was determined from the objective function value (OFV), goodness-of-fit diagnostics, percent relative standard error (%RSE), conditional number, and assessments of covariate impact on steady-state exposure. Interindividual variability was assumed to follow a log-normal distribution and separate log-additive residual error terms were estimated for serial and sparse sampling conditions.

#### PK model covariate analysis

Prespecified covariates of demographic factors (age, sex, race, body weight, ethnicity), laboratory variables (estimated glomerular filtration rate (eGFR), albumin, total bilirubin, aspartate aminotransferase), and disease state (hyperlipidemia, type 2 diabetes mellitus, or healthy) were evaluated in a full covariate model. Continuous covariates were described using power models and categorical covariates were described using proportional shift models, as shown:

$${\theta }_{TV, ij }={\theta }_{REF}{\left(\frac{{x}_{ij}}{{x}_{REF}}\right)}^{{\theta }_{x}}$$

$${\theta }_{TV,ij}={\theta }_{REF}\cdot \left(1+{\theta }_{x}\cdot {x}_{ij}\right)$$

where *θ*_*REF*_ and *θ*_*x*_ are fixed-effect parameters and *x*_*ij*_ is the individual covariate value or indicator variable that is equal to 1 or 0. Covariate effects on apparent systemic clearance (CL/F) and apparent central distribution volume (Vc/F) were determined simultaneously. For covariate effects evaluated on bioavailability, the effect was interpreted as the relative bioavailability for the covariate test condition relative to the reference condition. Covariates that were poorly estimated or had a small effect on bempedoic acid PK parameter predictions (i.e., estimate < 0.1 or ratio of estimate/standard error ≤ 2) were removed from the model.

A stepwise backward-elimination procedure was used to remove covariates characterized by a change in OFV < 10.8 units (α = 0.001 for 1 degree of freedom). An ad hoc covariate analysis was performed using a forward-selection procedure to assess the impact of concomitant medications (atorvastatin, pravastatin, rosuvastatin, simvastatin, metformin, ezetimibe) on the bempedoic acid absorption rate constant (*K*_*a*_), F1, CL/F, and Vc/F parameters. At each step of the forward selection, covariates with the largest decrease in OFV below a cutoff of 10.8 units (*p* = 0.001 for 1 degree of freedom) were included in the ad hoc model, and the resulting model was termed the final model.

#### Model evaluation

At all stages of model development, standard model diagnostic plots were generated to provide a visual assessment of the model fit, including goodness-of-fit, concordance, residual, and random-effect distribution plots. The covariance matrix of estimates was inspected to verify that the extreme pairwise correlations (*p* >|0.95|) of the parameters were not encountered. Condition numbers of correlation matrices of parameter estimates (i.e., the ratio of the largest to smallest eigenvalues) were assessed to ensure values did not exceed 1000, which can be indicative of an ill-conditioned model [25].

The final model was evaluated using an internal visual predictive check (VPC) [26]. Simulated datasets (*n* = 1000) were conditioned by the design, population, dose regimen, sample size, and covariate distribution of the observed dataset. Summary measures were calculated for the median and 5th and 95th percentiles (90% confidence interval (CI)). The visual assessment of the predictive performance of the model was conducted by comparing the 5th and 95th percentiles of the simulated data against the observed 5th, 50th, and 95th percentiles of bempedoic acid concentrations binned by time.

#### Covariate effects on bempedoic acid PK

The impact of covariates retained in the final PK model were evaluated on steady-state bempedoic acid area under the curve (AUC_SS_). The significance of the results was summarized using forest plots generated by incorporating multiple levels of uncertainty and variability, including variation in covariate values and their correlation among individuals in the population, uncertainty in model parameter estimation, and variation in PK parameters among individuals. To maintain the correlation between individual covariates, 100 simulation datasets with the same number of individuals as the PK analysis set were generated by re-sampling of complete covariate vectors (i.e., unique participants) with replacement. One hundred sets of population fixed-effect parameter estimates were generated using a parametric bootstrapping procedure to account for uncertainty in the final parameter estimates. Each unique set of population parameters was paired with a unique dataset and used to simulate individual predictions (IPRED) of bempedoic acid AUC_SS_ to account for variation in individual PK parameters. Participants were grouped by covariate condition and mean AUC_SS_ in each group, as well as the AUC_SS_ ratio of test-to-reference group determined for each iteration of the simulation. The mean (90% CI) was derived as the 50th (5th, 95th) percentiles of the resulting distribution of 100 mean AUC_SS_ and AUC_SS_ ratio values.

### PopPK/PD analysis

#### Base model development

The development of the popPK/PD model followed a sequential approach, where the fitting of the popPK/PD model was conditioned on individual post hoc PK parameters estimated using the popPK model. Estimation of the popPK/PD model parameters was based on the observed LDL-C data and patient-level PK information derived from individual empirical Bayes estimates of the PK parameters. Data from patients and treatment periods with < 80% compliance were excluded from all modeling. For patients without observed PK data, their individual covariate vectors were included in the model and population-predicted (PRED) bempedoic acid concentrations were used in the popPK/PD model analysis.

A type 1 indirect response model [27] for serum LDL-C, with bempedoic acid inhibition of serum LDL-C production, was selected as the popPK/PD model, based on knowledge of the mechanism of action of bempedoic acid and previously reported model structures describing the relationship between statin exposure and LDL-C effect [28–30]. The rate of LDL-C change was determined according to the expression:

$$\frac{{dLDL}_{C}}{dt}={k}_{in}*\left[1-\frac{{I}_{max}*C}{{IC}_{50}+C}\right]-{k}_{out}*{LDL}_{C}$$

where response production is described by an apparent zero order rate constant (*k*_*in*_) and a nonlinear maximal effect model parameterized with maximum (fractional) inhibitory effect (I_max_), bempedoic acid concentration (C), and bempedoic acid concentration associated with 50% of I_max_ (IC_50_). LDL-C loss is described by the first-order rate constant (*k*_*out*_) and LDL-C concentration (LDL_C_), with LDL-C turnover represented as the inverse of *k*_*out*_ (1/*k*_*out*_). At baseline prior to drug exposure, steady-state conditions prevail (*dLDL*_*C*_*/dt* = 0) and *k*_*in*_ is determined as the product of *k*_*out*_ and baseline LDL-C. Residual error was estimated using an additive and proportional error model.

#### PD model covariate analysis

Prespecified covariates included demographic factors, creatinine clearance, disease state (HeFH or type 2 diabetes mellitus), observed baseline LDL-C, prior established LMTs, and concomitant medication (low-, moderate-, or high-intensity statin or ezetimibe). Covariate effects on baseline LDL-C and maximal drug effect were determined. The relationship between continuous or categorical covariates and the typical value of popPK/PD parameters was modeled as described above for the popPK analysis. The full model was subsequentially reduced by removing covariate-parameter relationships with a low magnitude of effect to facilitate covariate selection using Wald’s Approximation Method (WAM) [31]. The WAM procedure ranks all 2^*k*^ possible sub-models derived from the presence or absence of the number of covariate parameters (*k*) in the full model. The WAM algorithm approximates the log-likelihood surface by a quadratic equation in the covariate effects based on the estimates and asymptotic variance–covariance matrix of the estimates from the full model fit. Maximizing Schwarz’s Bayesian Criterion (SBC) was used to rank all 2^*k*^ possible models and the top 15 ranked models were fit using NONMEM® to calculate the actual SBC.

#### Model evaluation

The approach to the popPK/PD model evaluation was identical to the strategy used to evaluate the popPK model described above. Visual assessment of the predictive performance of the popPK/PD model was conducted by comparing the 5th and 95th percentiles of the simulated data against the observed 5th, 50th, and 95th percentiles of LDL-C concentration data as a function of time and predicted bempedoic acid concentration.

#### Covariate effects on bempedoic acid PK/PD

The impact of the final popPK/PD model covariates on steady-state LDL-C achieved with treatment was assessed. Forest plots were generated by incorporating multiple levels of uncertainty and variability, including variation in covariate values and their correlation among individuals in the population, uncertainty in model parameter estimation, and variation in PK/PD parameters among individuals. To maintain the correlation between individual covariates, 100 simulation datasets with the same number of individuals as the PK/PD analysis set were generated by re-sampling of complete covariate vectors (i.e., unique patients) with replacement. One hundred sets of population fixed-effect parameter estimates were generated using a parametric bootstrapping procedure to account for uncertainty in the final parameter estimates. Each unique set of population parameters was paired with a unique dataset and used to simulate the IPRED of steady-state LDL-C response to account for variation in individual PK/PD parameters. Patients were grouped by covariate condition, and the mean LDL-C in each group was determined for each iteration of the simulation. The mean (90% CI) was derived as the 50th (5th, 95th) percentiles of the resulting distribution of 100 LDL-C values.

## Results

The PK dataset comprised 11,124 samples pooled from an intended target population of 2261 participants enrolled in 22 studies. Post-dose samples with concentrations below the limit of quantification (5.8%) and samples collected prior to the first dose of bempedoic acid were excluded from the dataset, resulting in a popPK analysis dataset containing 10,347 quantifiable PK samples from 2232 study participants. The popPK/PD model analysis included 27,534 LDL-C concentrations from 2984 patients who received bempedoic acid and 1475 placebo-controlled participants enrolled in 15 studies. Of the participants who received bempedoic acid, PRED concentrations were used in the popPK/PD analysis of 989 (33%) patients who did not have measurable plasma concentrations of bempedoic acid. A median LDL-C baseline of 113 mg/dL was observed for bempedoic acid–treated patients and 110 mg/mL for the placebo group, where both groups included participants who were receiving a stable regimen of LMTs at the time of study-treatment dosing on Day 1, as well as participants with no current ongoing LMT. Demographic and other characteristics were similar across the study participants included in the popPK and popPK/PD analyses (Table 1).

**Table 1:** Summary of demographic and baseline characteristics of study participants

Parameter	PopPK model	PopPK/PD model	
		Bempedoic acid	Placebo
Participants, *n*	2232	2984	1475
Sex, *n* (%)			
Male	1324 (59.3)	1899 (63.6)	938 (63.6)
Female	908 (40.6)	1085 (36.4)	537 (36.4)
Age, y			
Mean ± SD	60.5 ± 12.3	63.3 ± 10.2	63.9 ± 10.1
Median (range)	62.0 (18.0–89.0)	64.0 (21.0–91.0)	65.0 (20.0–88.0)
Body weight, kg			
Mean ± SD	85.1 ± 17.3	86.0 ± 17.3	85.9 ± 16.4
Median (range)	83.7 (42.5–152)	84.5 (42.5–160)	84.7 (39.2–170)
Baseline LDL-C (mg/dL)			
Mean ± SD	NA	122 ± 39.2	118 ± 38.2
Median (range)	NA	113 (48.0–422)	110 (38.0–411)
eGFR, mL/min^a^			
Mean ± SD	91.4 ± 24.5	90.4 ± 24.3	90.0 ± 23.6
Median (range)	89.3 (16.9–286)	88.1 (30.5–286)	87.5 (30.9–212)
Race, *n* (%)			
White	1978 (89.0)	2761 (92.5)	1349 (91.5)
Black	205 (9.2)	166 (5.6)	100 (6.8)
Asian	24 (1.0)	32 (1.1)	13 (0.9)
Native American	6 (0.2)	3 (0.1)	2 (0.1)
Native Hawaiian	6 (0.2)	6 (0.2)	3 (0.2)
Other^b^	13 (0.6)	16 (0.5)	8 (0.5)
Ethnicity, *n* (%)			
Not Hispanic	2025 (90.7)	2777 (93.1)	1179 (79.9)
Hispanic	207 (9.2)	207 (6.9)	124 (8.4)
Unknown	0	0	172 (11.7)
Disease state, *n* (%)			
Hyperlipidemia	1999 (89.6)	2916 (97.7)	1427 (96.7)
Diabetes^c^	359 (16.1)	631 (21.1)	333 (22.6)
Healthy^d^	184 (8.2)	18 (0.6)	6 (0.4)

*eGFR* estimated glomerular filtration rate, *HeFH* heterozygous familial hypercholesterolemia, *LDL-C* low-density lipoprotein cholesterol, *MDRD* modification of diet in renal disease, *NA* not applicable, *PD* pharmacodynamics, *PK* pharmacokinetics, *SD* standard deviation

^a^eGFR was calculated using the MDRD formula and expressed in absolute units (mL/min) without body surface area adjustment

^b^Included participants characterized as Mixed, Other, or Unknown race

^c^The popPK dataset included 49 patients with diabetes plus 310 patients with hyperlipidemia and diabetes. The popPK/PD dataset included 50 treated patients and 42 placebo patients with diabetes, plus 581 treated patients and 291 placebo patients with hyperlipidemia and diabetes

^d^A single study (Study 04) enrolled 24 healthy subjects and was included in the popPK/PD dataset

### PopPK model

**Fig. 1:**
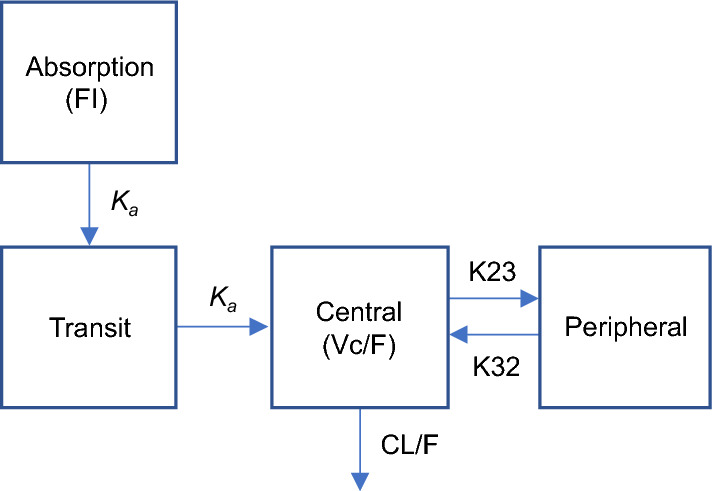
Bempedoic acid popPK model structure with two-compartment disposition and a single transit absorption compartment with linear elimination. *CL/F* apparent systemic clearance, *F1* oral bioavailability (typical value fixed at 1, such that systemic parameters are apparent), *K23* distribution rate constant (central to peripheral compartment), *K32* distribution rate constant (peripheral to central compartment), *K*_*a*_ absorption rate constant, *popPK* population pharmacokinetics, *Vc/F* apparent central distribution volume

The base popPK model was a two-compartment disposition model with a single transit absorption compartment and linear elimination (Fig. 1). Bempedoic acid concentration increased in proportion with the dose and no model parameters describing dose nonlinearity (i.e., dose-dependent bioavailability, nonlinear elimination) were required. The effect of food on absorption was evaluated as a structural covariate in the base model, where administration with food decreased the rate of bempedoic acid absorption but did not change the extent of absorption (Table 2).

**Table 2:** Final PopPK model parameters

Theta/parameter	Estimate	%RSE^a^	95% CI
CL/F, L/h	0.755	2.6	(0.716, 0.794)
Vc/F, L	19.1	6.9	(16.5, 21.7)
*K*_*a*_, h^−1^	1.41	5.6	(1.25, 1.56)
K23, h^−1^	0.184	8.3	(0.154, 0.215)
K32, h^−1^	0.156	4.3	(0.143, 0.169)
Food on *K*_*a*_	− 0.777	1.4	(− 0.799, − 0.754)
Covariates of CL/F			
Female sex	− 0.127	16.9	(− 0.169, − 0.0846)
Body weight	0.61	9.8	(0.493, 0.727)
Black race	− 0.143	18.0	(− 0.194, − 0.0924)
Hyperlipidemia	− 0.0945	28.4	(− 0.147, − 0.0416)
T2DM	− 0.177	16.7	(− 0.235, − 0.119)
eGFR	0.574	6.3	(0.503, 0.645)
Ezetimibe	− 0.0934	28.4	(− 0.146, − 0.0413)
Covariates of Vc/F			
Female sex	− 0.0895	33.4	(− 0.148, − 0.0308)
Age	0.743	15.9	(0.511, 0.976)
Body weight	0.94	20.8	(0.557, 1.32)
Simvastatin	− 0.154	29.2	(− 0.242, − 0.0654)
Covariates of F1^b^			
Atorvastatin	0.142	19.1	(0.0886, 0.195)
Residual error^a^, %			
Serial PK sampling	31.9	1.1	(31.2, 32.6)
Sparse PK sampling	54.3	1.3	(52.9, 55.7)
IIV, %CV			
CL/F	29.7		(27.7, 31.5)
Vc/F	100		(93.9, 106)
* K*_*a*_	73.9		(65.8, 81.2)
OFV	− 2725.975		

*%CV* percent coefficient of variation, *%RSE* percent relative standard error, *CL/F* apparent systemic clearance, *eGFR* estimated glomerular filtration rate, *F1* oral bioavailability, *IIV* interindividual variability, *K23* distribution rate constant (central to peripheral compartment), *K32* distribution rate constant (peripheral to central compartment), *K*_*a*_ absorption rate constant, *OFV* objective function value, *PK* pharmacokinetics, *T2DM* type 2 diabetes mellitus, *Vc/F* apparent central distribution volume

^a^Residual error and %RSE were represented as positive values by calculating the square root of (estimate)^2^

^b^F1, relative oral bioavailability was estimated for participants receiving concomitant atorvastatin relative to those not receiving concomitant atorvastatin

Initial attempts to evaluate covariate effects by adding all prespecified covariates simultaneously to the base model were unsuccessful. A working full model was subsequently identified by selectively removing covariates with a small magnitude of estimated effect and/or imprecise estimation. Key typical PK parameters in the working full model were precisely estimated with %RSE < 15% (data not shown).

A final model was subsequently identified following both a stepwise backward-elimination procedure for covariates included in the working full model and a forward-selection procedure for concomitant medications (Table 2). Covariates on CL/F that were retained included sex, body weight, race, hyperlipidemia, type 2 diabetes mellitus, and eGFR. Sex, age, and body weight were identified as statistically significant covariates on Vc/F. The covariates of ethnicity, albumin, total bilirubin, and aspartate aminotransferase did not satisfy the criteria for inclusion in the model. Ad hoc covariate analyses of concomitant medications identified three additional covariate effects for inclusion in the final model: atorvastatin on F1, simvastatin on Vc/F, and ezetimibe on CL/F. Goodness-of-fit diagnostic scatter plots revealed the model was consistent with observed data, although an underprediction of bempedoic acid occurred at high concentrations (Online Resource 2). However, PRED concentrations diverge from the line of unity at concentrations above 30–40 µg/mL, which reflect outlier maximum plasma concentration (C_max_) values greater than 2–3 standard deviations from the mean, where mean ± SD steady-state C_max_ at 180 mg/day was determined to be 20.6 ± 6.1 µg/mL [11]. Conditional and individual weighted residuals were generally well distributed across the range of predicted concentrations and time after dosing (Online Resource 2). Prediction-corrected VPC plots confirmed the ability of the popPK model to represent the central tendency of the observed bempedoic acid concentration–time data, with a large proportion of observed concentration data within the 90% prediction intervals of the model over a time horizon representative of steady-state (Online Resource 3). As shown in Table 2, key structural parameters (CL/F, Vc/F, *K*_*a*_) were well estimated in the final model, with %RSE < 10%. Interindividual variability was higher for Vc/F (100 percent coefficient of variation (%CV)) and *K*_*a*_ (73.9 %CV) than CL/F (29.7 %CV), likely reflecting sparse PK sampling early in the concentration–time course in phase 3 studies. Correspondingly, residual error was higher for studies with sparse sampling designs (54.3%) than those with more intensive sampling (31.9%).

**Fig. 2:**
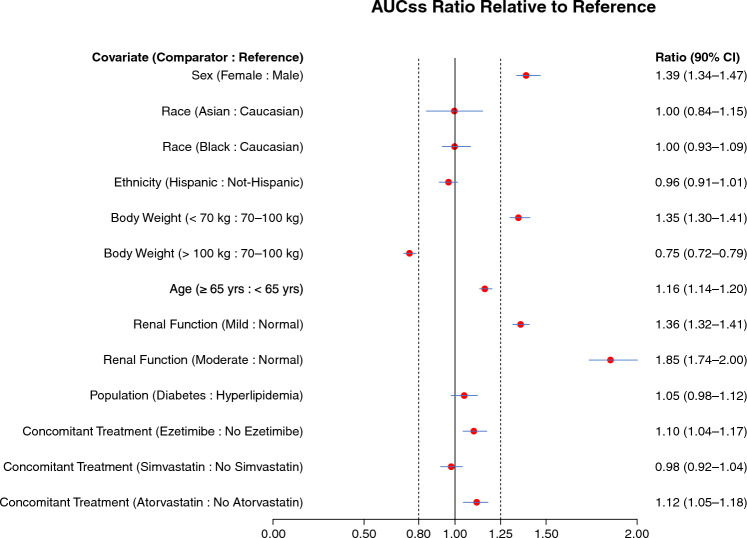
Influence of covariate populations on predicted bempedoic acid AUC_SS_. The median ratio of the typical parameter value in the test participant compared with the reference condition (red symbols) and corresponding 90% CI (blue line) is shown for each covariate. The AUC_SS_ ratios of test to reference at 0.8 and 1.25 are indicated by vertical dashed lines. *AUC*_*SS*_ steady-state area under the concentration–time curve from time zero to 24 h, *CI* confidence interval (Color figure online)

Simulations were performed using the final model to evaluate covariate effects using forest plots (Fig. 2). Renal impairment had the greatest effect on exposure predictions, with mild (eGFR 60 to < 90 mL/min) and moderate (eGFR 30 to < 60 mL/min) renal impairment predicted to increase AUC_SS_ by 1.36-fold (90% CI 1.32, 1.41) and 1.85-fold (90% CI 1.74, 2.00), respectively, relative to a population with normal renal function (eGFR ≥ 90 mL/min). In a phase 1 study to evaluate the safety, tolerability, and PK of a single 180 mg oral dose of bempedoic acid in study participants with mild, moderate, or severe renal impairment, no relationship was observed between the extent of renal impairment and number of adverse events, and no new safety signals were identified in participants with impaired renal function compared with those with normal renal function [23]. Women had 1.39-fold greater exposure (90% CI 1.34, 1.47) relative to men. Body weight was examined by approximate quartiles, which showed a modest inverse relationship between body size and predicted AUC_SS_. Lower body weight (Q1, < 70 kg) was predicted to increase AUC_SS_ by 1.35-fold (90% CI 1.30, 1.41) and higher body weight (Q4, > 100 kg) was predicted to decrease AUC_SS_ by 0.75-fold (90% CI 0.72, 0.79) compared with the interquartile range (Q2–Q3, 70–100 kg). The use of concomitant statins and/or ezetimibe did not meaningfully affect AUC_SS_ relative to participants not receiving these concomitant medications.

### PopPK/PD model

**Fig. 3:**
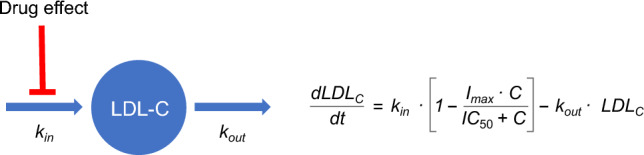
Indirect response popPK/PD model incorporating LDL-C turnover and empirical maximum inhibitory drug effect on the production rate of plasma LDL-C. *C* bempedoic acid concentration, *IC*_*50*_ concentration producing 50% of the maximum inhibitory effect, *max* maximum (fractional) inhibitory response, *LDL-C* low-density lipoprotein cholesterol, *k*_*in*_ zero-order rate constant representing LDL-C production, *k*_*out*_ first-order rate constant describing LDL-C elimination, *popPK/PD* population pharmacokinetics/pharmacodynamics

The relationship between bempedoic acid exposure and LDL-C lowering was adequately characterized by an indirect popPK/PD response model incorporating LDL-C turnover and an empirical maximum inhibitory drug effect on the production rate of plasma LDL-C (Fig. 3). Parameter estimates and covariate effects of the final popPK/PD model are summarized in Table 3. Covariate effects on I_max_ that were retained in the model included sex, body weight, race, statin intensity, ezetimibe use, and prior statin therapy. Statin intensity, HeFH, diabetes, and prior statin or ezetimibe therapy were identified as statistically significant covariates of baseline LDL-C. Goodness-of-fit plots showed that the model fit of the observed data was adequate based on concordance between observations and population- and individual-model predictions with random scatter around the line of unity and no trends in residuals vs. model predictions or time indicative of systematic bias (Online Resource 4).

**Table 3:** Final popPK/PD model parameters

Theta/parameter	Estimate	%RSE	95% CI
PD parameters			
I_max_	0.350	5.20	(0.314, 0.386)
IC_50_, µg/mL	3.17	17.7	(2.07, 4.26)
Baseline LDL-C, mg/dL	143	0.800	(141, 145)
TURN, h	85.8	9.30	(70.2, 101)
Covariates of I_max_			
Ezetimibe	0.190	20.3	(0.114, 0.266)
Low-intensity statin	− 0.238	18.8	(− 0.325, − 0.150)
Moderate-intensity statin	− 0.302	7.50	(− 0.346, − 0.257)
High-intensity statin	− 0.424	4.50	(− 0.461, − 0.387)
Female sex	0.203	16.5	(0.137, 0.269)
Body weight	0.544	12.6	(0.410, 0.679)
Black race	− 0.240	19.1	(− 0.330, − 0.150)
Statin prior therapy	− 0.373	20.7	(− 0.525, − 0.222)
Covariates of baseline LDL-C			
Low-intensity statin	− 0.159	10.0	(− 0.191, − 0.128)
Moderate-intensity statin	− 0.268	2.80	(− 0.282, − 0.253)
High-intensity statin	− 0.293	2.40	(− 0.307, − 0.279)
HeFH	0.0671	27.0	(0.0316, 0.103)
T2DM	− 0.0661	13.0	(− 0.0830, − 0.0492)
Ezetimibe prior therapy	− 0.0596	27.5	(− 0.0917, − 0.0274)
Statin prior therapy	− 0.296	8.40	(− 0.345, − 0.247)
Residual error^a^			
PD proportional, %	15.3	0.900	(15.0, 15.6)
PD additive, g/dL	3.94	9.10	(3.24, 4.64)
IIV, %CV			
I_max_	43.1		(40.8, 45.3)
Baseline LDL-C	23.9		(23.4, 24.5)

*%CV* percent coefficient of variation, *%RSE* percent relative standard error, *CI* confidence interval, *HeFH* heterozygous familial hypercholesterolemia, *I*_*max*_ maximum (fractional) inhibitory response, *IC*_*50*_ concentration producing 50% of the maximum inhibitory effect, *IIV* interindividual variability, *LDL-C* low density lipoprotein cholesterol, *PD* pharmacodynamic(s), *PK* pharmacokinetic(s), *popPK/PD* population pharmacokinetics/pharmacodynamics, *T2DM* type 2 diabetes mellitus, *TURN* turnover time for LDL-C

^a^Residual errors are represented as positive values by calculating the square root of (estimate)^2^

The final popPK/PD model parameters were well estimated with good precision (Table 3). Bempedoic acid IC_50_ was estimated to be 3.17 µg/mL and I_max_ was estimated at 35% reduction in serum LDL-C from baseline. In absolute terms, the baseline LDL-C was estimated at 143 mg/dL, and the typical time required to achieve steady-state LDL-C concentrations following daily bempedoic acid dosing was approximately 3 weeks (99% of steady-state in 18 days), based on the model-predicted LDL-C turnover of 85.8 h. Covariates in the final popPK/PD model were selected using the WAM procedure, as the correlation between rank ordering of covariates by the WAM-predicted and NONMEM-estimated model was acceptable (r = 0.7). The predicted I_max_ values for bempedoic acid were decreased by Black race and statin treatment at baseline or with concomitant bempedoic acid. The predicted I_max_ values were increased by concomitant ezetimibe treatment, female sex, and body weight (Table 3).

**Fig. 4:**
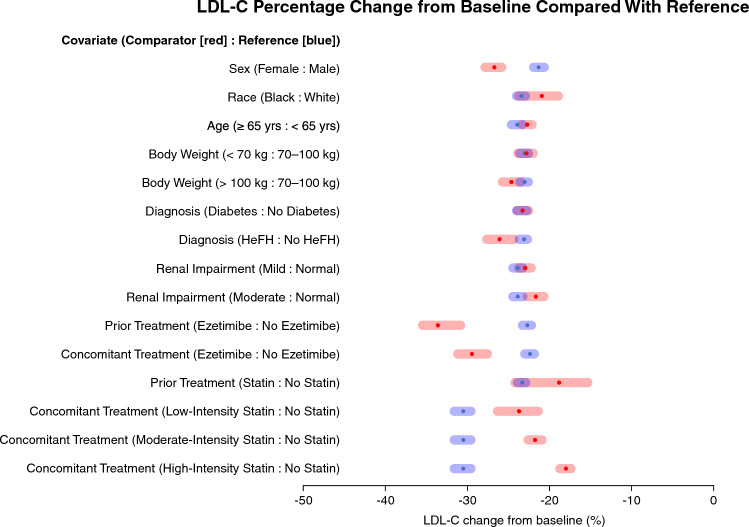
Influence of patient factors on the predicted LDL-C change from baseline in the popPK/PD model. The mean LDL-C level across all patients with the comparator (red) and reference (blue) covariate conditions of interest was calculated and summarized across 100 simulations to derive 90% CIs. *CI* confidence interval, *HeFH* heterozygous familial hypercholesterolemia, *LDL-C* low-density lipoprotein cholesterol, *popPK/PD* population pharmacokinetics/pharmacodynamics (Color figure online)

A bempedoic acid 180 mg once-daily regimen was predicted to result in a 28% median reduction in serum LDL-C from baseline. In addition, model-based predictions were generated for LDL-C at steady-state to assess the impact of the above intrinsic and extrinsic factors on the LDL-C response to bempedoic acid in the PK/PD population from 15 clinical studies (Fig. 4). Patient sex impacted LDL-C response. Specifically, females had a − 26.7% (90% CI − 27.8%, − 25.8%) LDL-C change from baseline compared with − 21.3% (90% CI − 21.9%, − 20.6%) for males when all other model covariates remained constant. Patients receiving concomitant ezetimibe treatment with bempedoic acid had a − 29.4% (90% CI − 31.2%, − 27.6%) change in LDL-C from baseline compared with − 22.4% (90% CI − 23.0%, − 21.8%) for those not receiving ezetimibe. Patients on low-intensity (− 23.7%; 90% CI − 26.3%, − 21.4%), moderate-intensity (− 21.8%; 90% CI − 22.6%, − 20.9%), or high-intensity (− 18.0%; 90% CI − 18.7%, − 17.4%) statin therapy in combination with bempedoic acid had a lower magnitude of LDL-C change from baseline compared with − 30.5% (90% CI − 31.6%, − 29.6%) for those receiving bempedoic acid without concomitant statin therapy. However, absolute LDL-C levels achieved at steady state were lower for all combinations, reflecting the impact of prior statin treatment on baseline LDL-C (Online Resource 5).

## Discussion

This study describes the development of popPK and popPK/PD models to characterize bempedoic acid, an ATP-citrate lyase inhibitor approved for the treatment of hypercholesterolemia. A popPK model was developed to perform a model-based meta-analysis of bempedoic acid PK during clinical development in phase 1, 2, and 3 studies. The time course of bempedoic acid concentrations after once-daily oral administration of 60–240 mg doses was adequately described by a two-compartment disposition model with a single transit absorption compartment and first-order elimination. The final popPK model predictions of bempedoic acid disposition PK parameters were CL/F = 0.755 L/h, Vc/F = 19.1 L, K23 = 0.184 h^−1^, and K32 = 0.156 h^−1^ for a typical study participant, with model covariates assigned to reference values. The alpha (distribution) and beta (elimination) phase half-lives corresponding to these parameters were 1.91 h and 40.7 h, respectively. The PK properties described by the model supported the bempedoic acid once-daily dosing regimen.

Food was predicted to decrease the transit rate of bempedoic acid absorption by 78% without affecting the extent of absorption. Several statistically significant covariate effects on the PK parameters of CL/F (sex, body weight, race, disease state, renal function, ezetimibe), Vc/F (sex, age, body weight, simvastatin), and F1 (atorvastatin) were identified in the popPK analysis. Point estimates for the exponents quantifying the relationship between body weight on CL/F (0.61) and Vc/F (0.94) are concordant with the values assumed in theory-based allometry (0.75 for CL/F, 1 for Vc/F) [32].

An indirect popPK/PD response model was developed to link decreases in LDL-C levels with bempedoic acid concentrations, where bempedoic acid acted as an inhibitor of response production. Based on the known impact of statins on LDL-C synthesis and degradation, initial model exploration evaluated the effect of bempedoic acid on *k*_*in*_ inhibition and *k*_*out*_ stimulation [33]. During model development, parameter estimates with greater numerical stability were obtained when drug effect was placed on *k*_*in*_ inhibition. In addition, concordance between the model-derived estimate of *k*_*out*_ (0.3 day^−1^) and the fractional clearance rate observed in steady-state tracer turnover studies (0.306–0.380 day^−1^) provided further confidence in the system-based model estimates [33, 34]. The estimate of *k*_*out*_ for bempedoic acid was also consistent with model-derived estimates of *k*_*out*_ for statins (0.2–0.3 day^−1^) [29, 30] and the PCSK9 inhibitors evolocumab (0.3 day^−1^) [35] and alirocumab (0.1 day^−1^) [36]. However, LDL-C data from bempedoic acid phase 3 studies are minimally informative to characterize the dynamic response of LDL-C to bempedoic acid treatment, as the first LDL-C sample (post baseline) was collected on Day 29 at near steady-state conditions. Although ATP-citrate lyase inhibition leads to LDL receptor upregulation, the expression levels of LDL receptors were not measured or accounted for by the popPK/PD model. Therefore, a semi-mechanistic indirect-response model based on cholesterol synthesis inhibition that incorporated LDL-C turnover with inhibitory bempedoic acid effects on plasma LDL-C concentrations was used.

The model predictions of LDL-C lowering, as percent change from baseline, were consistent with the overall treatment effect of bempedoic acid at a dose of 180 mg, where the observed average steady-state concentration of 12.5 μg/mL was approximately 3.9-fold greater than the IC_50_ value estimated by the model (3.17 µg/mL). At a steady-state bempedoic acid average concentration of 12.5 μg/mL, a 28% reduction in LDL-C from baseline was predicted by the model, accounting for approximately 80% of the predicted I_max_ at 35% maximal inhibition. At higher bempedoic acid doses, minimal additional decreases in LDL-C were predicted. This validated the conclusion that LDL-C changes established for the 180 mg dose were consistent with phase 3 study results, where a placebo-corrected least squares mean difference of a 27% reduction from baseline (with no background statin use) was observed, further confirming the robustness of the model [37]. A bempedoic acid 180 mg once-daily regimen was also predicted to provide optimal LDL-C lowering on a background of statin therapy, resulting in approximately 80% of the maximal achievable LDL-C reduction from baseline on stable statin therapy. PopPK/PD model simulations describing a median reduction in LDL-C of − 20% from baseline to week 12 in patients receiving maximally tolerated statin therapy with bempedoic acid (180 mg/day) accurately predicted an observed LDL-C lowering of − 16.5% from baseline to week 12 in a large phase 3 trial [6]. Moreover, the exposure–response relationship of bempedoic acid defined by the popPK/PD model indicates that PK changes resulting from the effects of intrinsic covariates, such as renal impairment and body weight, are unlikely to have a significant impact on LDL-C lowering. These model predictions support the currently approved bempedoic acid dosing as the most appropriate regimen to provide therapeutic benefit across populations. However, it should be acknowledged that approximately one-third of the patients included in popPK/PD model development did not have observed PK data. There is a potential for some underprediction of maximum concentrations using the PRED in these patients, as suggested by the goodness-of-fit plot for PRED concentrations (Online Resource 2).

While concomitant ezetimibe and statin treatments were identified as statistically significant covariates of bempedoic acid PK parameters, their impact on steady-state PK exposure is predicted to be minimal and not sufficient to warrant bempedoic acid dose adjustment. However, they still had significant covariate effects on bempedoic acid I_max_ for LDL-C. Concomitant use of ezetimibe was associated with an increase in I_max_, indicating greater LDL-C change from baseline with this combination, while statin use was linked to a reduction in bempedoic acid–mediated LDL-C lowering from baseline, with the magnitude of the effect displaying rank-order correlation with statin intensity. While the interaction between bempedoic acid and statins resulted in an inverse relationship between LDL-C percent change from baseline and statin intensity, steady-state absolute LDL-C levels were similar for moderate- and high-intensity combinations due to differences in baseline LDL-C in patients on stable statin therapy at study entry (Online Resource 5). These model predictions of concomitant use of statins with bempedoic acid to lower LDL-C are consistent with the effects of statins working through a common pathway of hepatic cholesterol synthesis inhibition, while ezetimibe acts through an independent pathway to block intestinal cholesterol absorption [38]. A published dose–response model for the effect of combining bempedoic acid with statins suggests a likely PD effect. However, the magnitude of the effect also reflects the combined different relative efficacies of bempedoic acid and statins, expressed as proportional terms, such as percent change from baseline [39]. Nevertheless, significant beneficial LDL-C lowering relative to baseline was observed across all statin intensities and the addition of bempedoic acid to a stable statin regimen increases the probability of achieving the LDL-C goals outlined in current guidelines [1, 2]. Absolute reductions in LDL-C were not meaningfully different between the moderate- and high-intensity statin groups when combined with bempedoic acid, and the model-predicted LDL-C–lowering effects of bempedoic acid (180 mg/day), when added to a stable statin regimen, were largely consistent with observed phase 3 clinical trial results [5–8, 12].

Covariate analysis identified associations between bempedoic acid PK and the intrinsic covariates of renal function, sex, and body weight. The covariate with the greatest impact on bempedoic acid PK was renal impairment. Higher exposures were reflected in the ratio of AUC_SS_ (90% CI) for mild (1.36 (1.32, 1.41)) and moderate (1.85 (1.74, 2.00)) renal impairment relative to participants with normal renal function. However, these increases are not expected to result in substantial LDL-C reduction; thus, no dose adjustment is warranted in this population based on efficacy and clinical safety data, indicating good tolerability of bempedoic acid without major exposure-related safety concerns.

Body weight was also identified as a covariate of bempedoic acid PK. The magnitude was modest, with participants with a lower body weight (< 70 kg) predicted to have a 1.35-fold higher exposure than those in the 70–100 kg range, while those with a higher body weight (> 100 kg) predicted to have a 0.75-fold lower exposure than those in the 70–100 kg range. However, these exposure differences are not predicted to impact LDL-C lowering. Covariate analysis of the popPK/PD model also identified a small effect on LDL-C lowering due to body weight. This small effect is consistent with a statin study on plaque reduction, in which a modestly smaller LDL-C percent reduction was achieved in patients with greater body mass index (at or above the study median) vs. those with lower body mass index [40].

Model-based predictions identified a greater percentage change from baseline in LDL-C for females (− 26.7%) compared with males (− 21.3%). These findings are congruent with the clinical data from the CLEAR Harmony trial, which determined a 5 percentage point estimated difference in LDL-C lowering from baseline in women vs. men with bempedoic acid [6, 12]. Female sex was associated with a 1.4-fold increase in bempedoic acid exposure. Higher exposures are anticipated to lead to small changes in efficacy. However, once exposure differences were accounted for, there was still an increase in the estimated maximum LDL-C–lowering benefit from bempedoic acid for females relative to males, suggesting a possible PD effect. The difference in LDL-C lowering observed between males and females could be in part due to the larger proportion of the latter group receiving low-intensity statin treatment in the phase 3 trials, as well as due to the impact that statin intensity has on LDL-C reduction from bempedoic acid. The trend observed in this analysis might also be related to additional mechanisms responsible for lipid metabolism, not just those involved in bempedoic acid metabolism [41, 42]. The results of this study are in contrast to those of the LIPID-REAL registry, which showed that the mean percent reduction in LDL-C was significantly smaller in women than men treated with PCSK9 inhibitors [43]. There appears to be some impact of bempedoic acid treatment on LDL-C lowering in female patients in this analysis, which is consistent with the clinical results. Nonetheless, the magnitude of the observed effect attributable to bempedoic acid is not clear.

## Conclusion

Pooled data from across the clinical development program for bempedoic acid were used to develop models to describe the PK and LDL-C lowering based on plasma exposure to bempedoic acid as well as to assess the influence of intrinsic and extrinsic covariates as sources of variability. In the population covariate analysis, reduced renal function, female sex, and body weight had a meaningful impact on steady-state exposure, but are not expected to significantly affect the LDL-C–lowering effect of bempedoic acid. The model predicted that a bempedoic acid dose of 180 mg/day achieved an effect on LDL-C that was near the plateau of the exposure–response curve, supporting the adequacy of this dose regimen. While administration of bempedoic acid on a background of statin therapy resulted in a smaller percent change from baseline than monotherapy, significant LDL-C lowering was observed across all statin intensities, with statin intensity having no meaningful effect on the efficacy profile. In comparison, administration with ezetimibe increased the overall LDL-C–lowering effect, a PD effect resulting from the independent LDL-C–lowering pathway of ezetimibe. This analysis suggests women may have slightly better LDL-C lowering from bempedoic acid compared with men. Age and race were not clinically significant covariates with respect to bempedoic acid PK or its effects on LDL-C lowering. Based on model-predicted responses, an adjustment of the bempedoic dose based on intrinsic and extrinsic factors is not warranted.

## Supplementary Information

Below is the link to the electronic supplementary material.

Supplementary file1 (DOCX 1384 KB)

## Data Availability

At this time, the data, analytical methods, and study materials will not be made available to other researchers for the purposes of reproducing the results or replicating the procedures.
