# Supplementary material for: Population pharmacokinetic and pharmacokinetic-pharmacodynamic modeling of bempedoic acid and low-density lipoprotein cholesterol in healthy subjects and patients with dyslipidemia
Source: J Pharmacokinet Pharmacodyn. 2023 May 27;50(5):351–64. doi: 10.1007/s10928-023-09864-w (PMC10460718; doi:10.1007/s10928-023-09864-w)
Supplement: Supplementary file 1 — Supplementary file1 (DOCX 1384 KB) [file 10928_2023_9864_MOESM1_ESM.docx]

# Supplementary Information

**Population pharmacokinetic and pharmacokinetic-pharmacodynamic modeling of bempedoic acid and low-density lipoprotein cholesterol in healthy subjects and patients with dyslipidemia**

*Journal of Pharmacokinetics and Pharmacodynamics*

Satyawan B. Jadhav, Benny M. Amore, Howard Bockbrader, Ryan L. Crass, Sunny Chapel, William J. Sasiela, Maurice G. Emery

Address correspondence to:

**Benny M. Amore, PhD**

Senior Director, Esperion Therapeutics

Email: bamore@esperion.com

## Online Resource 1. Summary of studies included in the population pharmacokinetics and population pharmacokinetic/pharmacodynamic modeling analyses

| Study number^a^ | Phase | Design | Population | PK sampling scheme | LDL-C sampling scheme |
| --- | --- | --- | --- | --- | --- |
| 001^b^ | 1 | Randomized, double-blind, placebo‑controlled, crossover, escalating single-dose; bempedoic acid 2.5–250 mg or PBO | Healthy males and females  (*N* = 18) | Predose, 0.5, 1, 1.5, 2, 2.5, 3, 4, 6, 8, 12, 24, 48, 72, 96, 120, and 144 h postdose at beginning of each period and at EOS | — |
| 002  NCT01105598 | 1 | Randomized, double-blind, placebo‑controlled, ascending, multiple‑dose; cohorts 1–4: bempedoic acid 20–120 mg or PBO for 14 days; cohort 5: bempedoic acid 120 mg or PBO for 28 days | Males and females with mild dyslipidemia  (*N* = 56) | Cohorts 1–4: day 1: 4 and 8 h; days 2, 4, 6, and 11: trough; day 8: trough, 4 h; day 14: trough, 0.5, 1, 2, 3, 4, 6, 8, and 12 h; days 15, 17, and 22: morning; cohort 5: days 4, 8, 15, 22, and 28: trough | Cohorts 1–4: screening: twice with ≥ 7 days between each sample; days 1, 3, 8, and 15; cohort 5: days −1, 4, 8, 15, 22, and 28 |
| 003 [[1](#_ENREF_1)]  NCT01262638 | 2 | Randomized, double-blind, placebo‑controlled, parallel group; bempedoic acid 40, 80, or 120 mg or PBO for 12 weeks | Males and females with hypercholesterolemia  (*N* = 176) | Days 15, 29, 57, and 85: predose | Screening period: days −49 to −42; qualifying period: days −17 to −14 and days −10 to −7; days 1, 15, 29, 57, and 85 |
| 004  NCT01485146 | 1 | Randomized, double-blind, placebo‑controlled, ascending, multiple‑dose; bempedoic acid 140–220 mg or PBO for 14 days | Healthy males and females  (*N* = 24) | Day 1: 4 and 8 h postdose; days 2, 4, and 6: trough; day 8: trough and 4 h postdose; day 11: trough; day 14: trough and 0.5, 1, 2, 3, 4, 6, 8, and 12 h postdose; days 15, 17, and 22: morning | Screening and days 1, 4, 8, 15, and 22 |
| 005 [[2](#_ENREF_2)]  NCT01607294 | 2 | Randomized, double-blind, placebo‑controlled, parallel group; bempedoic acid 80 mg or PBO for 14 days, followed by bempedoic acid 120 mg or PBO for 14 days | Males and females with type 2 diabetes mellitus  (*N* = 60) | Days 7, 14, 15, 22, 28, and 29: trough; 4 h postdose on days 14 and 28 | Screening and days −1, 1 (prior to dosing), 4, 8, 15, 22, and 29 |
| 006 [[3](#_ENREF_3)]  NCT01751984 | 2 | Randomized, double-blind, placebo‑controlled, parallel group; 2‑week PBO run-in period, followed by bempedoic acid 60–240 mg for 8 weeks | Males and females with hypercholesterolemia who are statin intolerant  (*N* = 54) | Days 1, 15, 29, 43, and 57: predose | Screening and days 1, 15, 29, 43, and 57 |
| 007  NCT01779453 | 2 | Randomized, double-blind, placebo‑controlled, parallel group, drug interaction; atorvastatin 10 mg for 4 weeks, followed by bempedoic acid 60–240 mg for 8 weeks | Males and females with hypercholesterolemia  (*N* = 52) | Predose on days 14, 28, and 42; day 56: predose and 1, 2, 3, 4, 6, 8, 12, and 24 h postdose | Screening and days 1, 14, 28, 42, and 56 |
| 008 [[4](#_ENREF_4)]  NCT01941836 | 2 | Randomized, double-blind, parallel group; 5-week washout period, followed by bempedoic acid 120 or 180 mg, ezetimibe 10 mg, or bempedoic acid 120 or 180 mg + ezetimibe 10 mg for 12 weeks | Males and females with hypercholesterolemia, with or without being statin intolerant  (*N* = 322) | Predose on days 15, 29, 57, and 85 | Screening: weeks −6 and −1; days 1, 15, 29, 57, and 85 |
| 009 [[5](#_ENREF_5)]  NCT02072161 | 2 | Randomized, double-blind, placebo‑controlled, parallel group; washout of all lipid-lowering drugs except atorvastatin 10–2 mg, followed by bempedoic acid 120 or 180 mg or PBO for 12 weeks | Males and females with hypercholesterolemia receiving ongoing statin therapy  (*N* = 132) | Predose days 15, 29, 43, 57, and 85 | Screening: weeks −6, −2, and −1; days 1, 15, 29, 43, 57, and 85 |
| 012^b^ | 1 | Open-label, single-sequence, drug–drug interaction; stable baseline statin with bempedoic acid 240 mg | Healthy males and females  (*N* = 36) | Day −5: predose and 0.5, 1, 1.5, 2, 3, 4, 5, 6, 8, 10, and 12 h post statin dose; days −4 to 1: 18, 24, 36, 48, 72, 96, and 120 h post statin dose; days 10 to 11: predose; days 12 to 17: predose and 0.5, 1, 1.5, 2, 3, 4, 5, 6, 8, 10, 12, 18, 24, 36, 48, 72, 96, and 120 h post statin dose | — |
| 013 [[6](#_ENREF_6)] | 1 | Randomized, double-blind, placebo‑controlled, parallel group; metformin 500 mg alone for 28 days, followed by metformin 500 mg + bempedoic acid 180 mg for 14 days | Males and females with type 2 diabetes mellitus  (*N* = 30) | Days 11 and 12: predose; day 14: predose and 0.5, 1, 2, 3, 4, 6, 8, 12, 16, and 24 h post metformin + bempedoic acid dose | Screening: days −35, −10, −7 (optional), −1; 1, 4, 8, and 14 |
| 014  NCT02178098 | 2 | Randomized, double‑blind, placebo‑controlled, parallel group; washout of lipid-lowering agents and antihypertensives; run-in of PBO for 3 weeks, followed by bempedoic acid 180 mg or PBO for 6 weeks | Males and females with elevated LDL-C and hypertension  (*N* = 144) | Days 15, 29, and 43: predose | Screening/washout: weeks −6, and −1; days 1, 15, 29, and 43 |
| 023^b^ [[7](#_ENREF_7)] | 1 | Open-label, single-dose, parallel group; single dose of bempedoic acid 180 mg | Males and females who have normal kidney function or some degree of renal impairment  (*N* = 24) | Day 1: predose and 1, 2, 3, 4, 6, 8, 12, and 24 h postdose; and days 3, 4, 5, 6, 7, 8, 10, 12, 14, 16, and 18 postdose | — |
| 032^b^ [[8](#_ENREF_8)] | 1 | Nonrandomized, open-label, single-dose, parallel group; single dose of bempedoic acid 180 mg | Males and females who have normal liver function or some degree of hepatic impairment  (*N* = 24) | Day 1: predose, 0.5, 1, 2, 3, 4, 6, 8, 10, 12, 24, 48, 72, 96, 120, 144, 168, 192, 216, and 240 h postdose | — |
| 035^b^ [[9](#_ENREF_9)]  NCT02659397 | 2 | Randomized, double-blind, placebo‑controlled, parallel group, open‑label atorvastatin 80 mg + PBO or bempedoic acid 180 mg for 4 weeks | Males and females who are treated with statins  (*N* = 60) | Days 8, 14, 15, 21 and 29: predose | — |
| 037^b^ | 1 | Open-label, drug–drug interaction, single‑sequence; one of four assigned statins for 5 days, followed by coadministration of statin with bempedoic acid 180 mg for 12 days | Healthy males and females  (*N* = 48) | Day −5: predose and 0.5, 1, 1.5, 2, 3, 4, 5, 6, 8, 10, and 12 h post statin dose; days −4 to 1: 18, 24, 36, 48, 72, 96, and 120 h post statin dose; days 10 to 11: predose; days 12 to 17: predose and 0.5, 1, 1.5, 2, 3, 4, 5, 6, 8, 10, 12, 18, 24, 36, 48, 72, 96, and 120 h post statin dose | — |
| 039 [[10](#_ENREF_10)]  NCT03193047 | 2 | Randomized, double-blind, parallel group, placebo-controlled; washout of lipid‑lowering therapy; run-in PCSK9 inhibitor 420 mg for 3 months, followed by coadministration of PCSK9 inhibitor and bempedoic acid 180 mg or PBO | Males and females with hyperlipidemia  (*N* = 52) | Days 1, 31, and 61: predose trough | Days −135, −90, −60, −30, 1, 31, and 61 |
| 040 [[11](#_ENREF_11)]  NCT02666664 | 3 | Randomized, double-blind, placebo‑controlled, parallel group; bempedoic acid 180 mg vs. PBO 52 weeks | Males and females with high CV risk and hyperlipidemia  (*N* = 1950) | Days 85, 169, and 365: predose | Screening: week −2; days 1, 29, 57, 85, 169, 253, and 365 |
| 046 [[12](#_ENREF_12)]  NCT02988115 | 3 | Randomized, double-blind, placebo‑controlled, parallel group; bempedoic acid 180 mg or PBO added to existing lipid-lowering therapy for 24 weeks | Males and females with elevated LDL-C levels who are statin intolerant  (*N* = 300) | Days 1, 29, 85, and 169: predose | Days −35, −7, 1, 29, 85, and 169 |
| 047 [[13](#_ENREF_13)]  NCT02991118 | 3 | Randomized, double-blind, placebo‑controlled, parallel group; bempedoic acid 180 mg or PBO added to existing lipid-lowering therapy for 52 weeks | Males and females with hyperlipidemia at high CV risk not adequately controlled by current therapy  (*N* = 525) | Days 169 and 365: predose | Screening: weeks −5 and −1; days 1, 29, 85, 169, and 365 |
| 048 [[14](#_ENREF_14)]  NCT03001076 | 3 | Randomized, double-blind, placebo‑controlled, parallel group; run-in ezetimibe 10 mg, followed by coadministration of ezetimibe 10 mg and bempedoic acid 180 mg or placebo for 12 weeks | Males and females with elevated LDL-C levels  (*N* = 225) | Days 29, 57, and 85: predose | Days −35, −7, 1, 29, 57, and 85 or EOS |
| 055^b^ | 1 | Randomized, open-label, crossover; single-dose, fixed-dose combination | Healthy males and females  (*N* = 16) | Days 1 to 6: predose and 0.5, 1, 2, 3, 4, 5, 8, 10, 12, 24, 48, 72, 96, and 120 h postdose | — |

^a^Study number with reference and ClinicalTrials.gov identifier, if applicable

^b^Studies included in popPK analysis only. Remaining 15 studies were included in both popPK and popPK/PD analyses
*CV* cardiovascular, *EOS* end of study, *IR* immediate release, *LDL-C* low-density lipoprotein cholesterol, *PBO* placebo, *PCSK9* proprotein convertase subtilisin/kexin type 9, *popPK* population pharmacokinetics, *popPK/PD* population pharmacokinetic/pharmacodynamic.

## Online Resource 2. Population PK final model diagnostic plots. Observed bempedoic acid concentration versus individual-predicted (A; IPRED) and population-predicted (B; PRED) bempedoic acid concentration; Time versus conditional weighted residuals (C; CWRES) and individual weighted residuals (D; IWRES); PRED versus CWRES (E); and IPRED versus IWRES (F). Solid line represents the line of identity.


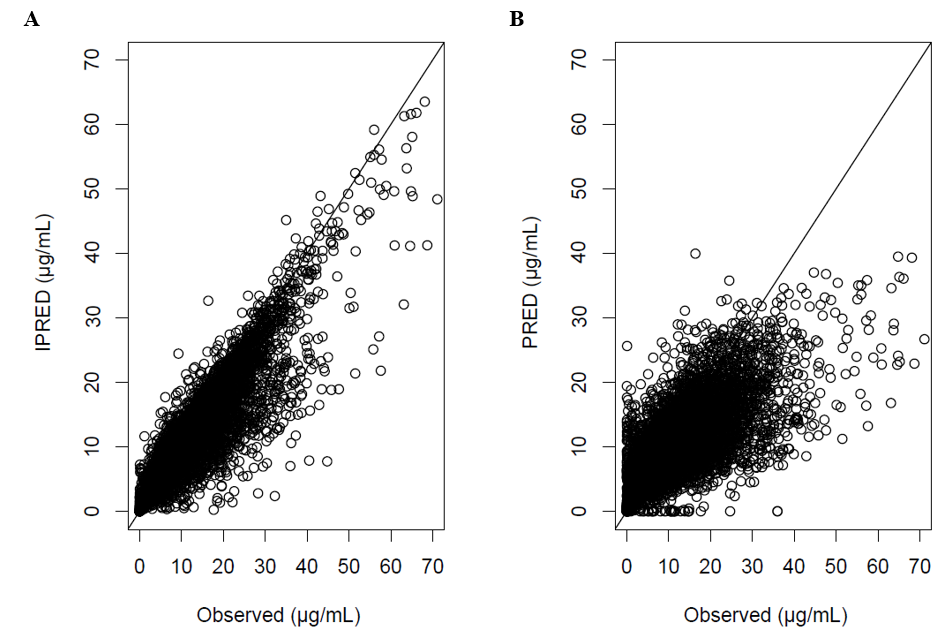


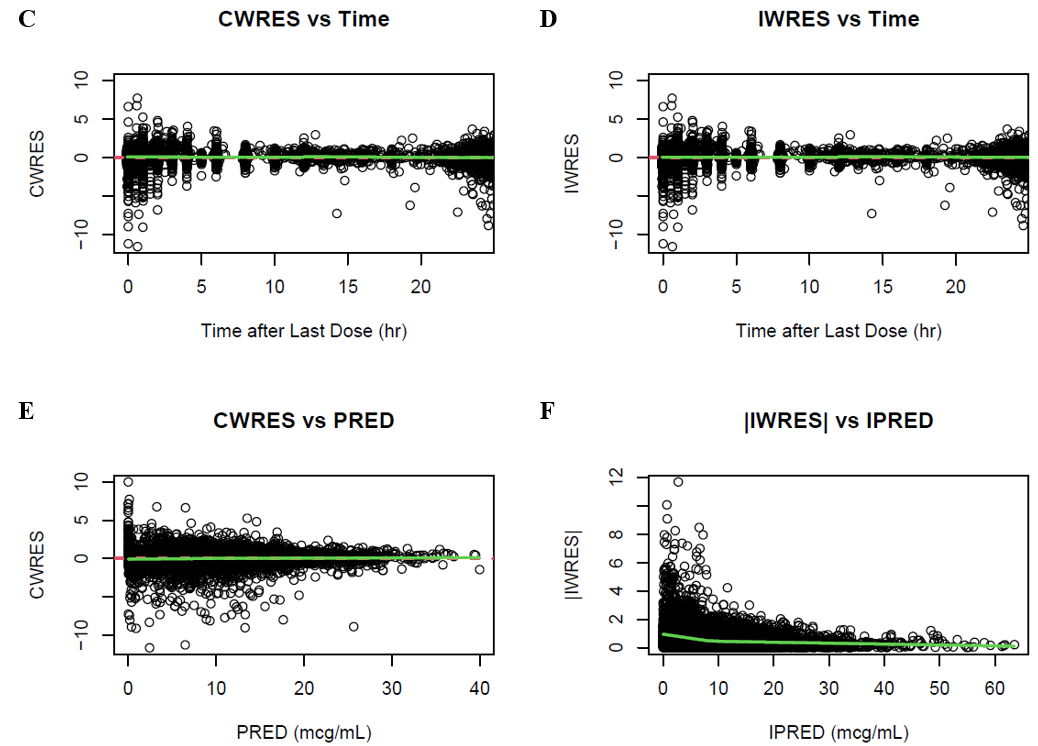


## Online Resource 3. Prediction-corrected visual predictive check of bempedoic acid concentrations binned by nominal day over one month (A) and one year (B) in all participants treated with once-daily bempedoic acid 2.5 mg to 240 mg administration from 22 clinical trials.


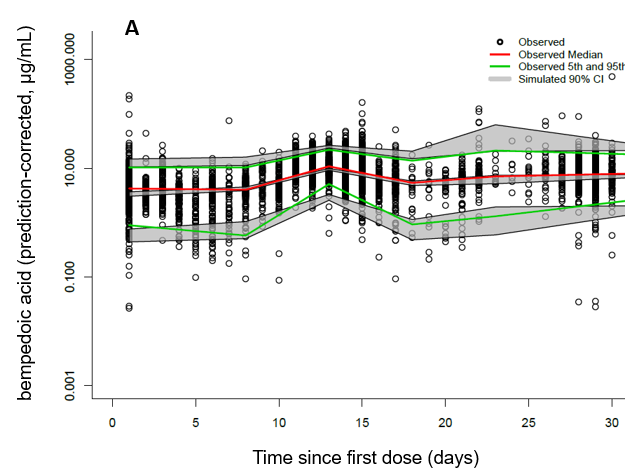

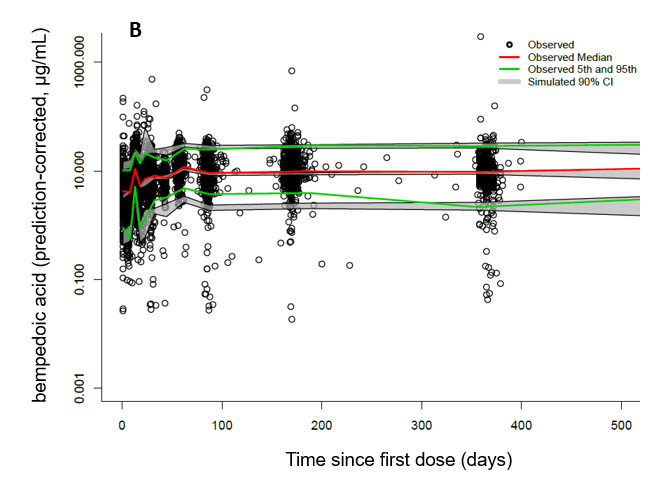


## Online Resource 4. Population PK/PD final model diagnostic plots. Observed LDL-C concentration (DV) versus individual-predicted (A; IPRED) and population-predicted (B; PRED) LDL-C concentration; Time (C) and PRED (D) versus weighted residuals (WRES); Time (E) and PRED (F) versus conditional weighted residuals (CWRES); IPRED versus individual weighted residuals (G; IWRES). Solid line represents the line of identity.


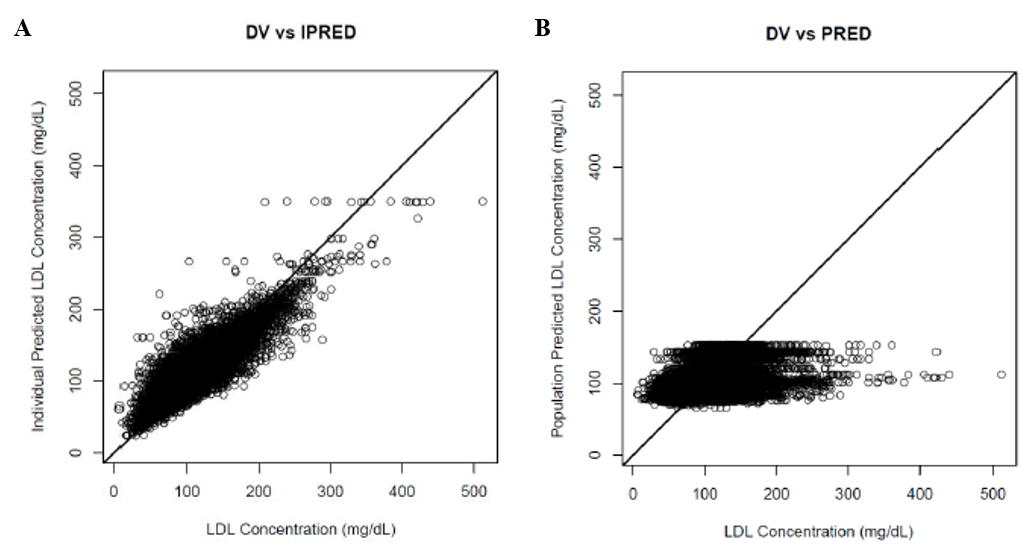

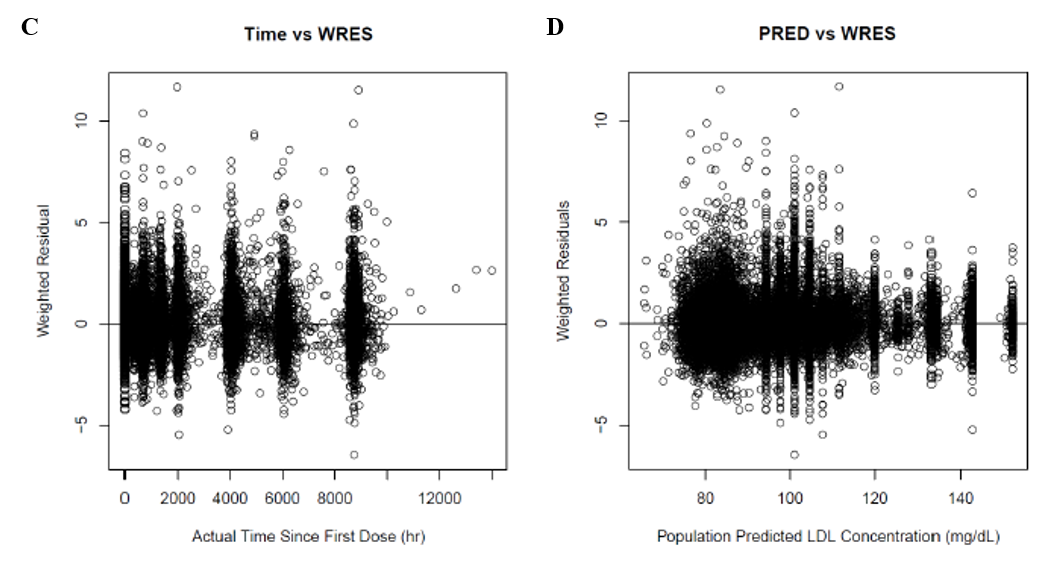

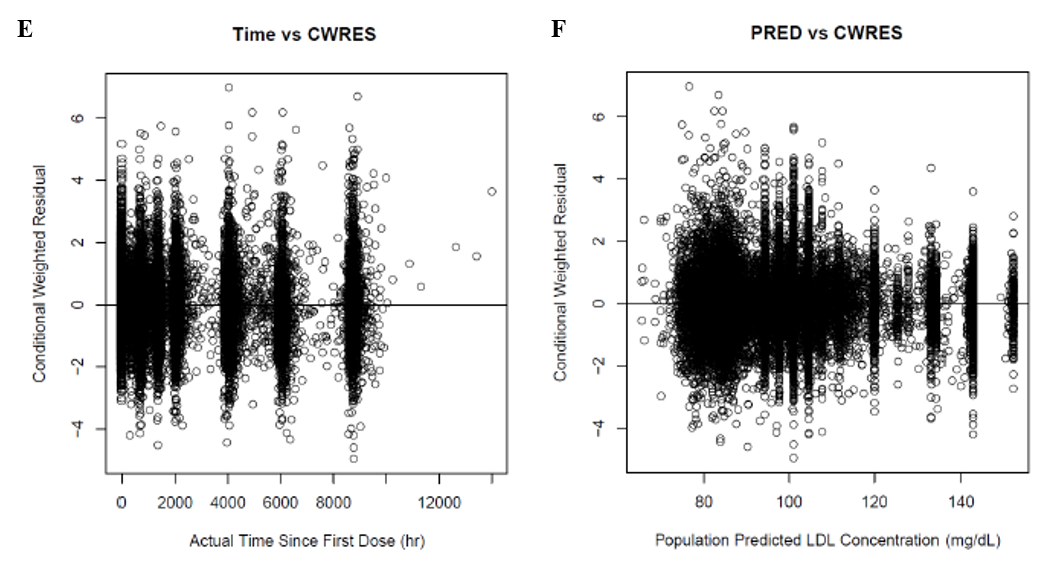

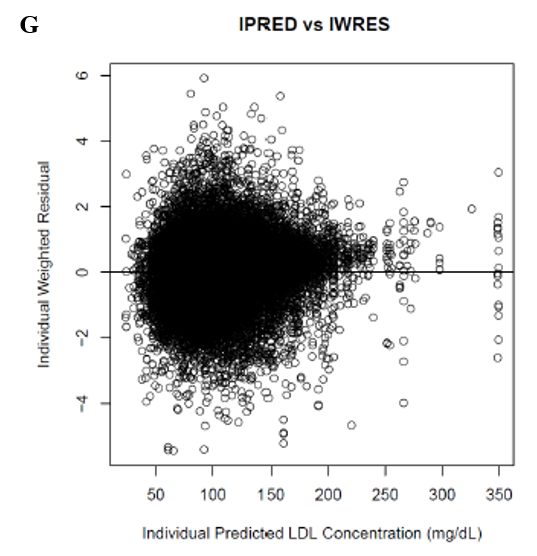


## Online Resource 5. Model-based predictions of absolute LDL-C and LDL-C change from baseline using the popPK/PD model

| **Description** | **Comparator** | **Reference** | **Comparator** | **Reference** |
| --- | --- | --- | --- | --- |
| **Covariate (Comparator : Reference)** | **LDL-C (90% CI) [mg/dL]** | **LDL-C (90% CI) [mg/dL]** | **LDL-C CFB (90% CI) [% ]** | **LDL-C CFB (90% CI) [% ]** |
| Sex (Female : Male) | 87.9 (86.48–89.42) | 89.68 (88.74–90.76) | −26.71 (−27.84; −25.81) | −21.32 (−21.92; −20.64) |
| Race (Black : White) | 99.48 (95.67–104.22) | 88.36 (87.32–89.36) | −20.93 (−23.7; −18.92) | −23.39 (−24; −22.93) |
| Age (≥ 65 yrs : < 65 yrs) | 87.15 (86.33–88.53) | 90.86 (89.59–92.36) | −22.7 (−23.3; −22.12) | −23.92 (−24.62; −23.33) |
| Body Weight (< 70 kg : 70–100 kg) | 90.52 (88.78–92.63) | 89.13 (88.02–90.18) | −22.78 (−23.84; −21.99) | −23.02 (−23.61; −22.56) |
| Body Weight (> 100 kg : 70–100 kg) | 87.35 (85.6–88.65) | 89.13 (88.02–90.18) | −24.63 (−25.72; −23.52) | −23.02 (−23.61; −22.56) |
| Diagnosis (Diabetes : No Diabetes) | 83.25 (81.59–85) | 90.61 (89.4–91.96) | −23.27 (−23.92; −22.53) | −23.29 (−24; −22.77) |
| Diagnosis (HeFH : No HeFH) | 97.38 (92.95–101.73) | 88.53 (87.6–89.63) | −26.07 (−27.64; −24.35) | −23.08 (−23.69; −22.68) |
| Renal Impairment (Mild : Normal) | 88.77 (87.33–90.08) | 89.51 (88.49–90.9) | −22.95 (−23.73; −22.23) | −23.86 (−24.45; −23.24) |
| Renal Impairment (Moderate : Normal) | 87.6 (85.75–89.79) | 89.51 (88.49–90.9) | −21.65 (−22.71; −20.69) | −23.86 (−24.45; −23.24) |
| Prior Treatment (Ezetimibe : No Ezetimibe) | 85.07 (80.92–88.55) | 89.27 (88.3–90.34) | −33.58 (−35.44; −30.82) | −22.69 (−23.28; −22.2) |
| Concomitant Treatment (Ezetimibe : No Ezetimibe) | 86.06 (83.56–89.08) | 89.52 (88.5–90.52) | −29.44 (−31.17; −27.56) | −22.37 (−23; −21.84) |
| Prior Treatment (Statin : No Statin) | 84.06 (76.05–90.39) | 89.1 (88.12–90.13) | −18.83 (−24.2; −15.34) | −23.32 (−23.9; −22.88) |
| Concomitant Treatment (Low-Intensity Statin : No Statin) | 93.08 (88.37–97.3) | 99.47 (97.12–101.47) | −23.7 (−26.32; −21.37) | −30.49 (−31.58; −29.56) |
| Concomitant Treatment (Moderate-Intensity Statin : No Statin) | 83.18 (81.69–84.38) | 99.47 (97.12–101.47) | −21.76 (−22.6; −20.9) | −30.49 (−31.58; −29.56) |
| Concomitant Treatment (High-Intensity Statin : No Statin) | 83.99 (82.94–85.72) | 99.47 (97.12–101.47) | −18 (−18.73; −17.39) | −30.49 (−31.58; −29.56) |

*CFB* change from baseline, *CI* confidence interval, *LDL-C* low-density lipoprotein cholesterol, *PD* pharmacodynamics, *popPK* population pharmacokinetics, *yrs* years.

**Supplementary References**

1. Ballantyne CM, Davidson MH, Macdougall DE, Bays HE, Dicarlo LA, Rosenberg NL, Margulies J, Newton RS (2013) Efficacy and safety of a novel dual modulator of adenosine triphosphate-citrate lyase and adenosine monophosphate-activated protein kinase in patients with hypercholesterolemia: results of a multicenter, randomized, double-blind, placebo-controlled, parallel-group trial. J Am Coll Cardiol 62(13):1154-1162. <https://doi.org/10.1016/j.jacc.2013.05.050>.

2. Gutierrez MJ, Rosenberg NL, Macdougall DE, Hanselman JC, Margulies JR, Strange P, Milad MA, McBride SJ, Newton RS (2014) Efficacy and safety of ETC-1002, a novel investigational low-density lipoprotein-cholesterol-lowering therapy for the treatment of patients with hypercholesterolemia and type 2 diabetes mellitus. Arterioscler Thromb Vasc Biol 34(3):676-683. <https://doi.org/10.1161/atvbaha.113.302677>.

3. Thompson PD, Rubino J, Janik MJ, MacDougall DE, McBride SJ, Margulies JR, Newton RS (2015) Use of ETC-1002 to treat hypercholesterolemia in patients with statin intolerance. J Clin Lipidol 9(3):295-304. <https://doi.org/10.1016/j.jacl.2015.03.003>.

4. Thompson PD, MacDougall DE, Newton RS, Margulies JR, Hanselman JC, Orloff DG, McKenney JM, Ballantyne CM (2016) Treatment with ETC-1002 alone and in combination with ezetimibe lowers LDL cholesterol in hypercholesterolemic patients with or without statin intolerance. J Clin Lipidol 10(3):556-567. <https://doi.org/10.1016/j.jacl.2015.12.025>.

5. Ballantyne CM, McKenney JM, MacDougall DE, Margulies JR, Robinson PL, Hanselman JC, Lalwani ND (2016) Effect of ETC-1002 on serum low-density lipoprotein cholesterol in hypercholesterolemic patients receiving statin therapy. Am J Cardiol 117(12):1928-1933. <https://doi.org/10.1016/j.amjcard.2016.03.043>.

6. Hanselman JC, MacDougall DE, Amore BM, McGonigal J, Sasiela WJ, Emery MG (2020) Bempedoic acid effect on metformin pharmacokinetics and pharmacodynamics in patients with type 2 diabetes: In vitro-in vivo correlation. Clin Pharmacol Ther 107(Suppl 1):S43. Abstract PII-030. <https://doi.org/10.1002/cpt.1732>.

7. Amore BM, Sasiela WJ, Ries DK, Tresh P, Emery MG (2022) Pharmacokinetics of bempedoic acid in patients with renal impairment. Clin Transl Sci 15(3):789-798. <https://doi.org/10.1111/cts.13202>.

8. Amore BM, Sasiela WJ, Emery MG (2021) The effects of impaired hepatic function on pharmacokinetics of bempedoic acid, a first-in-class adenosine triphosphate (ATP) citrate lyase inhibitor, evaluated in an open-label, single-dose, parallel-group study. Clin Pharmacol Ther 109:S53.

9. Lalwani ND, Hanselman JC, MacDougall DE, Sterling LR, Cramer CT (2019) Complementary low-density lipoprotein-cholesterol lowering and pharmacokinetics of adding bempedoic acid (ETC-1002) to high-dose atorvastatin background therapy in hypercholesterolemic patients: A randomized placebo-controlled trial. J Clin Lipidol 13(4):568-579. <https://doi.org/10.1016/j.jacl.2019.05.003>.

10. Rubino J, MacDougall DE, Sterling LR, Kelly SE, McKenney JM, Lalwani ND (2021) Lipid lowering with bempedoic acid added to a proprotein convertase subtilisin/kexin type 9 inhibitor therapy: A randomized, controlled trial. J Clin Lipidol 15(4):593-601. <https://doi.org/10.1016/j.jacl.2021.05.002>.

11. Ray KK, Bays HE, Catapano AL, Lalwani ND, Bloedon LT, Sterling LR, Robinson PL, Ballantyne CM, CLEAR Harmony Trial (2019) Safety and efficacy of bempedoic acid to reduce LDL cholesterol. N Engl J Med 380(11):1022-1032. <https://doi.org/10.1056/NEJMoa1803917>.

12. Laufs U, Banach M, Mancini GBJ, Gaudet D, Bloedon LT, Sterling LR, Kelly S, Stroes ESG (2019) Efficacy and safety of bempedoic acid in patients with hypercholesterolemia and statin intolerance. J Am Heart Assoc 8(7):e011662. <https://doi.org/10.1161/jaha.118.011662>.

13. Goldberg AC, Leiter LA, Stroes ESG, Baum SJ, Hanselman JC, Bloedon LT, Lalwani ND, Patel PM, Zhao X, Duell PB (2019) Effect of bempedoic acid vs placebo added to maximally tolerated statins on low-density lipoprotein cholesterol in patients at high risk for cardiovascular disease: the CLEAR Wisdom randomized clinical trial. JAMA 322(18):1780-1788. <https://doi.org/10.1001/jama.2019.16585>.

14. Ballantyne CM, Banach M, Mancini GBJ, Lepor NE, Hanselman JC, Zhao X, Leiter LA (2018) Efficacy and safety of bempedoic acid added to ezetimibe in statin-intolerant patients with hypercholesterolemia: a randomized, placebo-controlled study. Atherosclerosis 277:195-203. <https://doi.org/10.1016/j.atherosclerosis.2018.06.002>.
